# Mechano-transduction in periodontal ligament cells identifies activated states of MAP-kinases p42/44 and p38-stress kinase as a mechanism for MMP-13 expression

**DOI:** 10.1186/1471-2121-11-10

**Published:** 2010-01-28

**Authors:** Nelli Ziegler, Angel Alonso, Thorsten Steinberg, Dale Woodnutt, Annette Kohl, Eva Müssig, Simon Schulz, Pascal Tomakidi

**Affiliations:** 1 Department of Oral Biotechnology, Dental School, University of Freiburg, Hugstetterstrasse 55, 79106 Freiburg, Germany; 2 Department of Orthodontics and Dentofacial Orthopaedics, Dental School, University of Heidelberg, Im Neuenheimer Feld 400, 69120 Heidelberg, Germany; 3 Division of Cell Differentiation, German Cancer Research Center, Im Neuenheimer Feld 242, 69120 Heidelberg, Germany; 4 Department of Orthodontics, Dental School, University of Freiburg, Hugstetterstrasse 55, 79106 Freiburg, Germany

## Abstract

**Background:**

Mechano-transduction in periodontal ligament (PDL) cells is crucial for physiological and orthodontic tooth movement-associated periodontal remodelling. On the mechanistic level, molecules involved in this mechano-transduction process in PDL cells are not yet completely elucidated.

**Results:**

In the present study we show by western blot (WB) analysis and/or indirect immunofluorescence (IIF) that mechanical strain modulates the amount of the matrix metalloproteinase MMP-13, and induces non-coherent modulation in the amount and activity of signal transducing molecules, such as FAK, MAP-kinases p42/44, and p38 stress kinase, suggesting their mechanistic role in mechano-transduction. Increase in the amount of FAK occurs concomitant with increased levels of the focal contact integrin subunits β3 and β1, as indicated by WB or optionally by IIF. By employing specific inhibitors, we further identified p42/44 and p38 in their activated, i.e. phosphorylated state responsible for the expression of MMP-13. This finding may point to the obedience in the expression of this MMP as extracellular matrix (ECM) remodelling executioner from the activation state of mechano-transducing molecules. mRNA analysis by pathway-specific RT-profiler arrays revealed up- and/or down-regulation of genes assigning to MAP-kinase signalling and cell cycle, ECM and integrins and growth factors. Up-regulated genes include for example focal contact integrin subunit α3, MMP-12, MAP-kinases and associated kinases, and the transcription factor c-fos, the latter as constituent of the AP1-complex addressing the MMP-13 promotor. Among others, genes down-regulated are those of COL-1 and COL-14, suggesting that strain-dependent mechano-transduction may transiently perturbate ECM homeostasis.

**Conclusions:**

Strain-dependent mechano-/signal-transduction in PDL cells involves abundance and activity of FAK, MAP-kinases p42/44, and p38 stress kinase in conjunction with the amount of MMP-13, and integrin subunits β1 and β3. Identifying the activated state of p42/44 and p38 as critical for MMP-13 expression may indicate the mechanistic contribution of mechano-transducing molecules on executioners of ECM homeostasis.

## Background

In addition to physiologic mechanical forces during swallowing, speaking or mastication the periodontal ligament (PDL) and its cells as part of the periodontium, i.e. the tooth holding apparatus is exposed to therapeutically applied forces, which aim at orthodontic tooth movement [1]. The PDL is a specialised soft connective tissue with viscoelastic properties, mainly comprised of fibroblasts and extracellular matrix (ECM) [2], among which the collagen type-I Sharpey fibers facilitate anchorage of the tooth in the alveolar bone [3].

The mechanical forces which interfere with the periodontium first address the PDL's ECM, thereby involving the PDL-fibroblasts (PDLF), since the cells are connected to the ECM by integrins [4]. Integrins as heterodimers consist of promiscuous α/β-chain-combinations, e.g. αvβ1 or αvβ3, facilitating cell-matrix-interactions *via *the formation of focal contacts, which are located at focal adhesion sites [5]. Integrins as transmembrane molecules interconnect the PDLF's extracellular microenvironment with their cytoplasmatic proteins and are therefore mechano-sensors or mechano-perceptors, pivotal for conversion of mechanical into biochemical signals [6]. This is achieved by transposing the external signal to mechano-transducing molecules, co-localised together with integrins in the focal adhesion complex [7]. One of the key molecules in mechano-transduction is the focal adhesion kinase FAK/p125^FAK ^which becomes activated through phosphorylation at 6 - 8 tyrosin residues upon engagement of focal contact integrins by ECM ligands [8]. In previous studies on PDLF our own findings revealed that FAK/p125^FAK ^appears to be mechano-sensitive, since its activity was modulated in response to strain [9]. Further molecules which are key players in signal transduction and localised down-stream from FAK are the MAP-kinases ERK1 and 2, also known as p42/44, and the p38 stress kinase [10,11]. Recently published results add to the growing body of evidence that these kinases are not only cornerstones in signal transduction, i.e. the mediation of signals from the plasma membrane to the nucleus upon specific growth factor-ligand complex formation, but also equal in prominence concerning mechano-transduction. This is exemplified in a study on myocytes which demonstrated that ERK is rapidly activated upon strain and that p38 stress kinase appears to be the cross-talk partner of ERK in the biological context of myocyte phenotype modulation and differentiation [12].

Thus, equal in contribution, the plasma membrane-cytoplasm signal-/mechano-transduction leads to the activation of transcription factors preceding signal transport into the nucleus [13], which are responsible for the transcription of signal-/mechano-sensing genes. Among the plethora of transcription factors c-fos has been identified as mechano-sensitive [14-16]. In conjunction with c-jun, c-fos forms to the AP-1 transcription factor, the latter localised on the promoter of the matrix metalloproteinase- (MMP) 13 [17]. MMPs, such as MMP-13 which has a wide substrate range including various collagens, fibronectin and proteoglycanes, are responsible for cleavage of ECM molecules under physiological conditions. Thereby they contribute not only to ECM homeostasis, but also to therapeutic or pathologic situations. Concerning the therapeutic situation, orthodontic tooth movement induced by mechanical forces is not only going along with periodontal remodelling including bone resorption and formation at the sites of pressure and tension, respectively, but also with remodelling of the ECM [18]. In the ECM-steady state, homeostasis is reflected by the balance of ECM synthesis and degradation, whereas degradation in turn becomes balanced by the expression and activation of MMPs, which are counteracted by their specific tissue inhibitors, termed TIMPs [19].

In the present study, we show that one of the cellular responses upon mechanical strain is exemplified by modulation of the expression of the activated form of MMP-13, which on the mechanistic level is governed by the activity of MAP- and stress kinases p42/44, and p38, respectively.

## Results

### Modulation of focal adhesion integrin subunits and FAK/p125^FAK ^in response to strain

On the cellular level, mechano-transduction induced by integrin-matrix-interactions and mechanical forces starts at focal adhesions, which is one type of integrin-based adhesion complexes comprising mechano-sensitive signalling molecules such as p125^FAK ^[20,7]. Therefore, we were first interested whether mechanical strain leads to modulation of focal adhesion integrin subunits and p125^FAK^, facilitating intracellular conversion of the mechanical into a biochemical signal.

**Figure 1:**
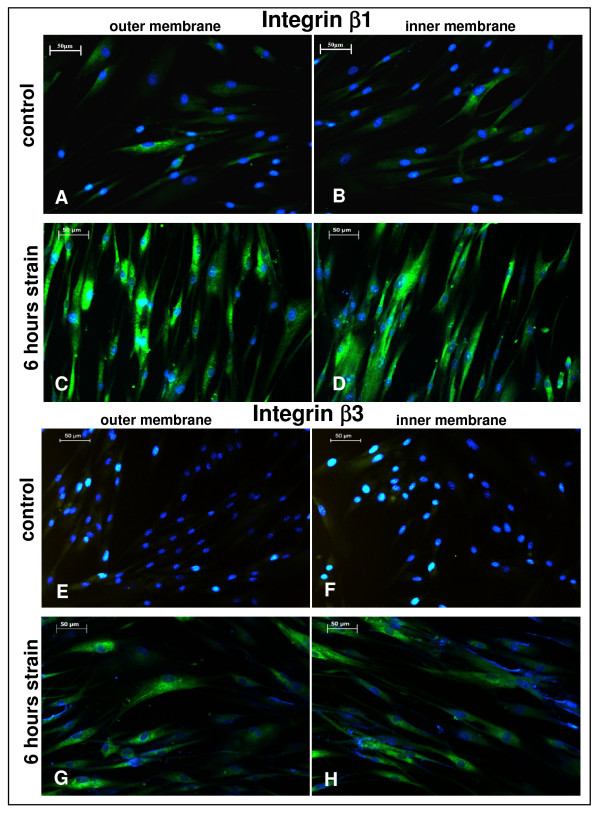
**Strain-induced integrin β1 and β3 expression in periodontal ligament (PDL) cells after 6 hours**. **(A) **PDL cells were seeded on flexible bottom cell culture dishes and strained with averaged 2.5% for 6 hours. For indirect immunofluorescence (IIF), the flexible membrane was divided into pieces derived from the inner and outer part of the membrane, and fixed for staining procedure. In IIF integrin subunit β1 **(A, B) **and subunit β3 **(E, F) **were visualized in controls and after 6 hours of strain-application (β1 **C, D **and β3 **G, H) **by using an anti-integrin β1 and β3-specific antibody. The green fluorescent signal illustrates the integrin β1 and β3 expression, while the blue fluorescence visualises the nuclear DAPI-staining. Scale bar, 50 μm.

Typical β-integrin subunits, located in focal adhesions are integrins β3 and β1, both of which in combination with αv bind to ECM constituents like fibronectin, vitronectin, and tenascin [21]. As exemplified by IIF for integrin β1, Figure 1 shows that control PDL cells which were not subjected to strain displayed discontinuous and faint β1-expression, regardless from whether the cells were growing at the outer (Figure 1A) or inner parts of the flexible membrane of the culture dish (Figure 1B). In contrast, the β1 subunit exhibited marked and homogenous expression when the PDLFs were exposed to mechanical strain for 6 hours, irrespective from membrane location (Figures 1C and 1D).

**Figure 2:**
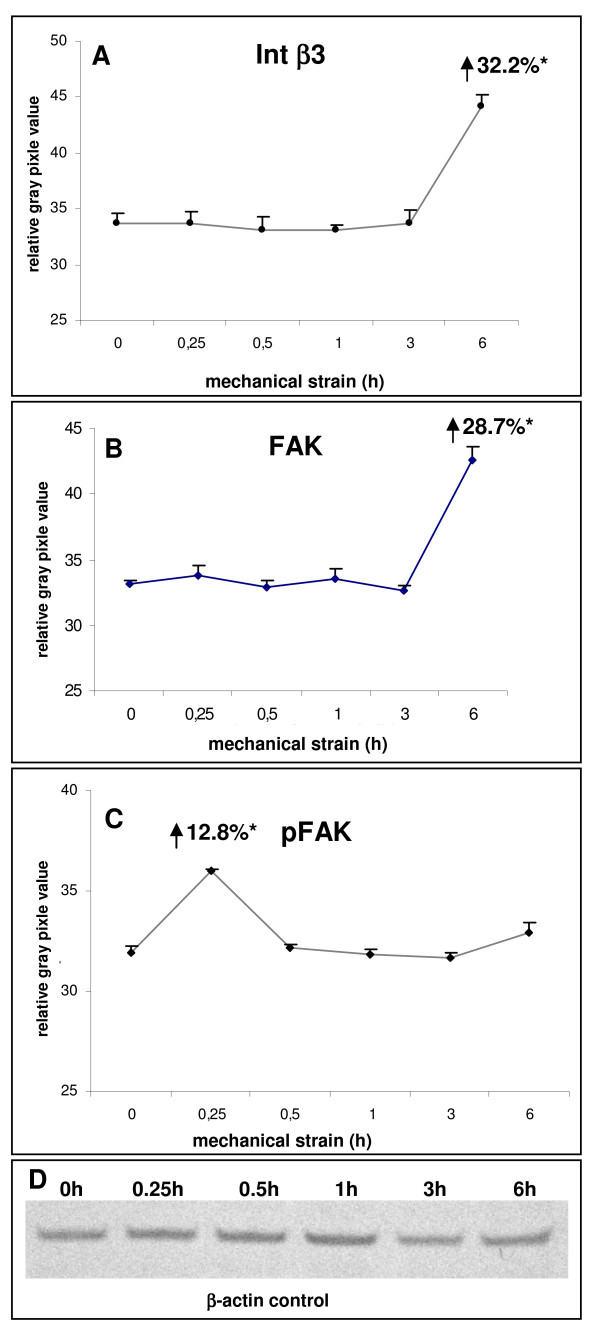
**Immunoblotting analysis of mechano-sensitive molecular constituents of the focal adhesion complex in PDL cells after strain-application**. PDL cells were seeded on flexible bottom cell culture dishes, strained with averaged 2.5% and followed by westernblot analysis. The numerical expression values were always denoted in relation to unstrained controls. **(A) **Expression levels of integrin β3 in PDL cells in response of the strain kinetic were detected with a mouse monoclonal anti-integrin β3 antibody and the maximum expression modulation was numerical notified in the graph after 6 h of strain. **(B) **For detection of the strain-induced total protein expression level of the focal adhesion kinase (FAK), a polyclonal rabbit anti-FAK antibody was used, and the maximum expression value was marked in the graph after 6 h strain. **(C) **Phosphorylation levels of Tyr^576 ^of FAK were assessed by immunoblotting using an antibody against phospho-Tyr^576^. The maximum activation status was numerically denoted after 0.25 h strain. **(D) **In addition, for loading control and for internal normalisation, β-actin was detected for each immunoblotting experiment. Data of each graph represent the mean of three individual experiments (n = 3), mean +/- SD and means were subjected to the Students T-test. All compared mean values with p < 0.01 were considered as statistical significant and are marked with an asterisk.

Despite its limitations in explanatory power concerning the amount and/or ratio of protein expression, the IIF results point to a strain-dependent obvious increase of expression for the β1 integrin subunit. This suggestion was supported by exploring β3 integrin, the further β subunit located in focal adhesions. By employing IIF and WB analysis as well, this increase appeared to be less pronounced for β3 with matched controls (Figures 1E, F and 1G, H). Coinciding with the IIF at 6 hours of strain, WB-analysis of β3 revealed an approximately 32% higher thus significant increase of protein expression with matched controls at this time point (Figure 2A).

After integrin-mediated mechano-perception at the plasma membrane or transmembrane site, mechanical loading intracellularly appeals focal adhesion kinase p125^FAK^, hereby leading to conversion into a biochemical signal. In the context of mechanical loading it has to be considered that mechano-transducing signalling proteins can be modulated in a dual fashion, including both, their total amount and their activation state. To address this issue adequately, we next investigated putative strain-associated modulation of p125^FAK ^as initiator of mechano-transduction both, on the total protein amount and its phosphorylation level. Concerning the total protein, the amount remained almost unchanged at the analysed time points of 15, 30 minutes, 1 and 3 hours, while after 6 hours of strain a drastic increase of 28.7% was noted (Figure 2B). Compared to the total protein amount, the phosphorylated, i.e. mechano-transducing active form of p125^FAK ^was modulated in a non-coherent fashion, as indicated by the significant gain in tyrosine phosphorylation of 12.5%, 15 minutes after strain induction (Figure 2C). At the later time points, the phosphorylation level appeared similar to non-strained control cells (Figure 2C). This finding supports the evidence that in PDL cells mechano-transduction occurs as early response to strain.

In the context of environmental cell-mechanosensing, the existence of a crosstalk between focal adhesions and the actin cytoskeleton should be noted [19]. Since in response to strain we have observed changes in the amount and/or activation of focal adhesion integrins and p125^FAK ^in PDL cells, we next focused on a possible actin modulation. With respect to this cytoskeletal constituent, almost equal protein bands were denoted at all strain periods under study (Figure 2D), strongly suggesting that the applied strain of averaged 2.5% has no impact on the actin amount in PDLF at the given strain periods and can therefore be applied for WB normalisation.

### Strain-associated mechano-transduction in PDL cells identifies activated states of p42/44 and p38 as a mechanism for MMP-13 expression

**Figure 3:**
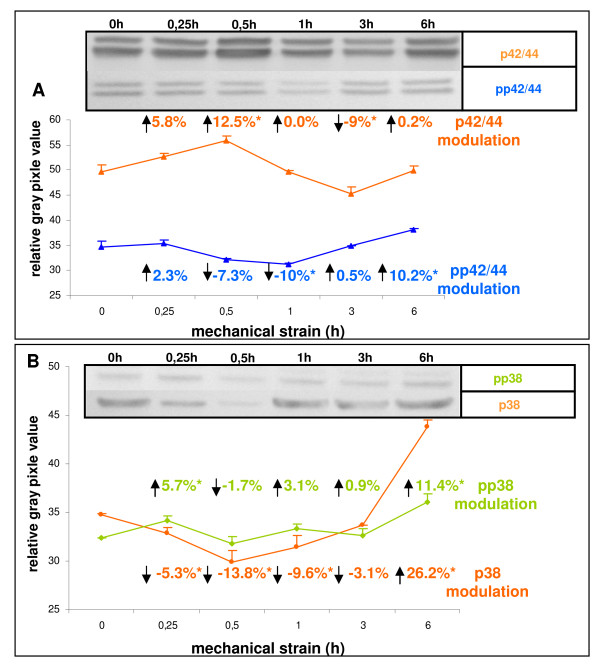
**Immunoblotting of molecules involved in mechano-signal-transduction**. PDL cells were seeded on flexible bottom cell culture dishes, strained with averaged 2.5% and followed by westernblot analysis. The numerical expression values were always denoted in relation to unstrained controls **(A) **The modulation of expression levels of the MAP-kinases p42/44 following strain application were detected with the monoclonal rabbit anti-p42/44 antibody, and the numerical changes of increase and decrease in expression were denoted by arrows in the graph. The phosphorylation levels of Thr^202^/Tyr^204 ^of p42/44 were assessed by immunoblotting, using an antibody against phospho- Thr^202^/Tyr^204^. The numerical changes in the activation status of pp42/44 in consequence of strain application were visualised by arrows in the graph. **(B) **The strain-induced expression levels of the MAP-kinase p38 were detected with a monoclonal rabbit anti-p38 antibody, and the expression changes increase or decrease were marked in the graph by arrows. The strain-induced activation status of phospho-p38 (pp38) was detected by immunoblotting using an antibody against phospho- Thr^180^/Tyr^182 ^of p38, and the modulation of expression levels was notified with numerical values and arrows in the graph. Data of each graph represent the mean of three individual experiments (n = 3), mean +/- SD and means were subjected to the Students T-test. All compared mean values with p < 0.01 were considered as statistical significant and are marked with an asterisk. The depicted western blots exemplify the protein expression changes of one biological replicate.

There is growing evidence from *in vitro *studies on various cell types that mechano-sensitive signalling molecules located down-stream from p125^FAK ^include MAP-kinases p42/44 and p38 stress-kinase [22,23]. Hence, we were further interested whether these signalling molecules render also targets of mechanical forces applied to PDL cells. Generally, all MAP-kinases exhibited strain-associated though non-coherent modulation with respect to the total protein amount and the phosphorylated, i.e. activated state, respectively, as shown in Figure 3. By connecting the given time-points of strain application, the resulting graphs of total protein and the phosphorylated state of p42/44 (Figure 3A, orange and blue graph), but also the p38 protein (Figure 3B, orange graph) displayed alternating courses, while that for p38 phosphorylation was oscillating (Figure 3B, green graph).

With matched controls, significant and therefore remarkable modulations were in detail seen for p42/44 at 30 minutes and 3 hours of strain, when the protein showed a 12.5% increase at 30 minutes and a 9.0% decrease in expression at 3 hours (Figure 3A, orange graph). Hallmarks in p42/44 phosphorylation were denoted at 1 and 6 hours of strain, with a decrease in activation of 10% at 1 and a nearly equal increase (10.2%) at 6 hours (Figure 3A, blue graph). For the protein amount of p38, strain application of 30 minutes revealed decrease of 13.8%, while at 6 hours increased levels of 26.2% were detected (Figure 3B, orange graph). Concerning the activated stress kinase situation, phosphorylation was up-regulated about 5.7% at 15 minutes, 3.1% at 1 hour, and 11.4% at 6 hours (Figure 3B, green graph). Although the behaviour of strain-induced modulation appeared divergent regarding the total protein amount and state of activation, the findings strongly suggest that the MAP-kinases under study are also key players in mechano-transduction in PDL cells. Considering the time periods of strain application, pp38 activation was stronger modulated at 15 minutes when compared to pp42/44 (compare Figure 3B, green graph, 5.7% for pp38 with Figure 3A, blue graph, 2.3% for pp42/44). This may be a hint that p38 is more sensitive concerning its mechano-responsivity at early stages of strain application.

Animal experiments conducted in rats support evidence that orthodontic tooth movement induced by mechanical forces involves the expression of proteases like MMP-13 on both the compression and tension site of the periodontium [24]. To further elucidate the presumed pivotal role of the studied MAP-kinases in mechano-transduction and MMP-13 as mechano-sensitive molecule in our PDL cells, we first investigated MMP-13 expression in response to strain application at the given time-periods. Then, the status of MMP-13 was analysed in conjunction with the respective MAP-kinase activity preceding pre-treatment of the PDL cells with inhibitors, which specifically address the phosphorylation of p42/44 and p38. Herewith, we wanted to ascertain whether MMP-13 expression mechanistically depends on mechano-transduction *via *the mentioned MAP-kinases.

**Figure 4:**
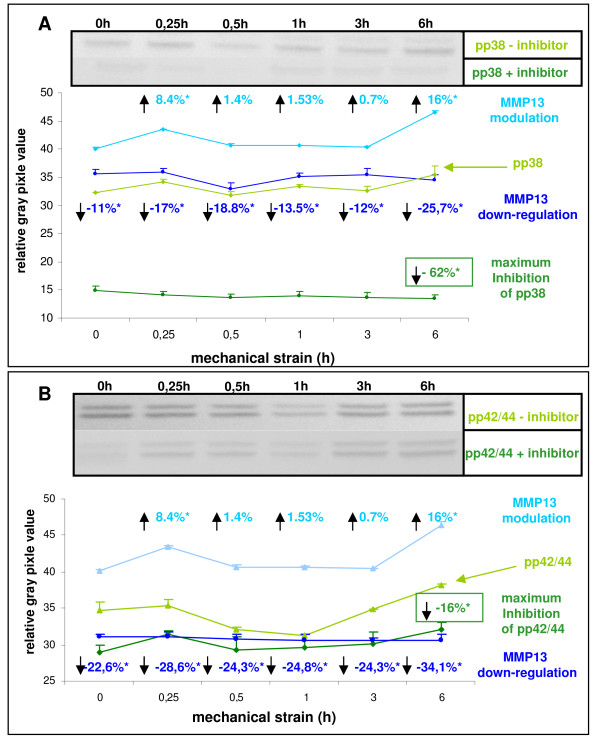
**Role of activated MAP-kinases pp38 and pp42/44 in strain-induced expression of the active form of MMP-13**. PDL cells were seeded on flexible bottom cell culture dishes, strained with averaged 2.5% and followed by westernblot analysis. The numerical expression values were denoted in relation to unstrained controls. The maximum inhibition was related to the respective strain-kinetic time points. (**A**) For strain-induced MMP-13 expression detection in a pp38-specific inhibition assay, PDL cells were pre-treated with 10 μM of a phosphorylation-specific inhibitor (SB202190) against pp38 and the MMP-13 down-regulation was compared *versus *untreated control. The numerical changes of the MMP13 protein expression status in consequence of strain application were visualised by arrows in the graph. Efficiency of pp38 inhibition yielded in a maximum inhibition of 62% after 6 hours strain and was visualised by the immunoblots (inset). (**B**) Strain-induced expression of MMP-13 in a pp42/44-specific inhibition assay was performed by pre-treating PDL cells with 10 μM of a phosphorylation-specific inhibitor (UO-126) against pp42/44 and the MMP-13 down-regulation was compared *versus *untreated control. The numerical changes of the MMP-13 protein expression status in consequence of strain application were visualised by arrows in the graph. Efficiency of pp42/44 inhibition yielded in a maximum inhibition of 16% after 6 hours strain and was visualised by the immunoblots (inset). Data of each graph represent the mean of three individual experiments (n = 3), mean +/- SD and means were subjected to the Students T-test. All compared mean values with p < 0.01 were considered as statistical significant and are marked with an asterisk. The depicted western blots exemplify the protein expression changes of one biological replicate.

Interestingly, MMP-13 expression by trend displayed similar strain-responsive modulation as seen before for the MAP-kinases, particularly for p38 stress-kinase. This is substantiated in Figure 4 by the marked levels of up-regulation of the protein amount of the activated form of MMP-13, being 8.4% at 15 minutes and 16% at 6 hours of strain (Figure 4A and Figure 4B, each with light blue). Pre-treatment of the PDL cells with the MAP-kinase-specific inhibitors generally yielded in a conspicuously pronounced decline of the protein abundance of the activated form of MMP-13, though this decline was stronger in case of pp42/44 inhibition. In detail, following inhibition of pp38 and pp42/44 as well the maximum decrease in the amount of MMP-13 was denoted at six hours of strain. While that for pp38 was approximately 26% (Figure 4A, dark blue graph) that seen for pp42/44 was about 34% (Figure 4B, dark blue graph). Irrespective from the degree of inhibition, these findings strongly suggest that the activated MAP-kinases states are truly mechano-responsive mechano-transducing elements and moreover they may render as a mechanism for MMP-13 expression in PDLF. Further, it appears noteworthy that at all studied strain periods MMP-13 decrease was more distinct upon pre-treatment with the pp42/44 inhibitor (Figure 4B, dark green graph) with matched pp38 inhibition (Figure 4A, dark green graph), although the repression of the MAP-kinases *per se *revealed a *vice versa *situation. Here, maximum inhibition for pp38 was 62% at 6 hours of strain (Figure 4A, dark green graph) while in case of pp42/44 it accounted 16% (Figure 4B, dark green graph).

This contrarious situation observed for the inhibition of MAP-kinase phosphorylation and decrease of MMP-13 expression on the other hand may point to a superior priority of the p42/44 mechano-/signal-transduction pathway in PDL cells.

In addition to MMPs, further executioners of ECM homeostasis are TIMPs which silence MMP-driven matrix degradation by their specific inhibition. Intriguingly, expression of TIMP-1, one of the most potent MMP inhibitors remained almost non-modulated in PDLF at any period of strain application (data not shown). These fairly constant levels observed for TIMP-1 juxtaposed with the strain-induced MMP-13 elevation support the hypothesis that mechanical forces may induce at least transiently a displacement of periodontal ECM homeostasis towards matrix degradation.

Being indicated in percentage, an overview of the protein amount of all analysed molecules or with respect to the MAP-kinases' degree of phosphorylation at each of the strain periods is given in Table 1.

**Table 1:** Expression and/or phosphorylation activation of proteins in PDL cells subjected to strain denoted in percentage

Molecule	control 0 h	0.25 h	0.5 h	1 h	3 h	6 h
integrin β3 (Int β3)	/	± 0	± 0	± 0	± 0	+32.2% * p < 0.01
focal adhesion kinase (FAK)	/	± 0	± 0	± 0	± 0	+ 28.7% * p < 0.01
phosphorylated focal adhesion kinase (pFAK)	/	+ 12.8% * p < 0.01	± 0	± 0	± 0	± 0
p42/44	/	+ 5.8% *	+ 12.5% * p < 0.01	± 0	- 9.0% * p < 0.01	+ 0.2% *
phosphorylated p42/44	/	+ 2.3% *	- 7.3% *	-10.0% * p < 0.01	+ 0.5% *	+ 10.2% * p < 0.01
phosphorylated p42/44 + inhibitor	/	/	/	/	/	maximum inhibition - 16.0% ⊗ p < 0.01
p38	/	- 5.3% * p < 0.01	- 13.8% * p < 0.01	- 9.6% * p < 0.01	- 3.1% *	+ 26.2% * p < 0.01
phosphorylated p38	/	+ 5.7% p < 0.01	- 1.7%	+ 3.1%	+ 0.9%	+ 11.4% p < 0.01
phosphorylated p38 + inhibitor	/	/	/	/	/	maximum inhibition - 62% ⊗
matrix metalloproteinase 13 (MMP13)	/	+ 8.4% * p < 0.01	+ 1.4% *	+1.53% *	+ 0.7% *	+ 16.0% * p < 0.01
matrix metalloproteinase 13 (MMP13) + pp38 inhibitor	- 11.0% ⊗ p < 0.01	- 17.0% ⊗ p < 0.01	- 18.8% ⊗ p < 0.01	- 13.5% ⊗ p < 0.01	- 12.0% ⊗ p < 0.01	- 25.7% ⊗ p < 0.01
matrix metalloproteinase 13 (MMP13) + pp42/44 inhibitor	- 22.6% ⊗ p < 0.01	- 28.6% ⊗ p < 0.01	- 24.3% ⊗ p < 0.01	- 24.8% ⊗ p < 0.01	- 24.3% ⊗ p < 0.01	-34.1% ⊗ p < 0.01

Densitometric measurements of western blot analysis were calculated in % of relative gray pixel values and normalised to control* or to the respective time point⊗ in the stretch kinetic. Date represent the mean of three independent experiments (n = 3) and means were subjected to the Students T-test. All compared mean values with p < 0.01 were considered as statistical significant.

### Mechanical strain modulates transcription of genes addressing biological functions such as MAP-kinase signalling and cell cycle, ECM and integrins, and growth factors

As indicated by WB analysis, in our PDL cells the most pronounced changes in the protein amount for β3 integrin as well as for MMP-13 and the mechano-sensitive signalling molecules FAK, p42/44, and p38 MAP-kinase were detected at 30 minutes and 6 hours of strain application. Hence, it was of interest to seek for putative strain- associated coincidences between protein and gene transcription alterations.

**Figure 5:**
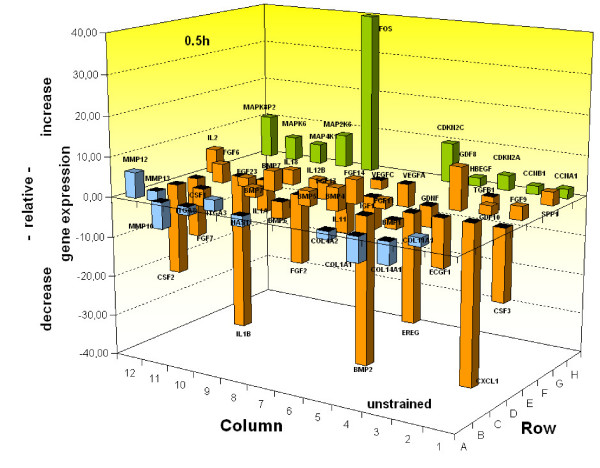
**Strain-induced quantitative mRNA-expression analysis of PDL cells related to MAP-kinase signalling and cell cycle, ECM and integrins, and growth factor pathways**. PDL cells were seeded on flexible bottom cell culture dishes and strained with averaged 2.5%. For quantitative mRNA analysis, PDL cells of unstrained controls and cells after 0.5 h strain period were harvested, and RNA was isolated and quantified for pathway-specific analysis. The relative increase and decrease in gene expression were internally normalised *versus *a quotient of 4 different housekeeping genes, and plotted in the graph. The data represent the mean of three independent experiments, and only significant expression modulations were included in the graph. The respective statistics were considered in the evaluation software. Plotted genes were allocated to a specific pathway and the respective columns were coloured for MAP-kinase signalling and cell cycle in orange, for ECM and integrins in light blue, and for growth factors in green, respectively.

By employing pathway-specific RT-profiler arrays, transcription of gene panels assigning to the biological functions (i) MAP-kinase signalling and cell cycle, (ii) ECM and integrins, and (iii) growth factors were quantitatively analysed. Scoring of gene transcription following a strain period of 6 hours revealed almost no changes in the above-mentioned pathway-specific RT-profilers, a finding, which also applied to the strain period of 3 hours (data not shown). Driven by these results, we concentrated on strain periods within 1 hour, thereby detecting significant transcriptional changes at 30 minutes with matched non-strained controls. Among these changes, significant up-regulation was denoted for 29 and significant down-regulation of transcription for 25 genes, which are illustrated for each pathway in Figure 5, and summarised together with their numerical factors in Table 2.

**Table 2:** Strain-induced pathway-specific quantitative mRNA-expression changes in PDL cells

gene symbol	relative gene expression value	gene symbol	relative gene expression value	gene symbol	relative gene expression value	gene symbol	relative gene expression value	gene symbol	relative gene expression value
***extracellular matrix and integrin pathway***		***growth factor pathway***		***growth factor pathway***		***growth factor pathway***		***MAP-Kinase signalling and cell cycle pathway***	
COL11A1	2.08	BMP1	-2.07	FGF11	2.14	GDF8	10.56	CCNA1	2.38
COL14A1	-5.43	BMP2	-36.76	FGF14	6.06	GDNF	-5.28	CCNB1	2.07
COL1A1	-6.23	BMP4	5.66	FGF17	4.00	IGF1	-8.00	CDKN2A	3.73
COL4A2	-2.06	BMP5	3.73	FGF2	-17.15	IL11	-10.20	CDKN2C	2.14
HAS1	-2.53	BMP6	-4.29	FGF22	5.10	IL12B	2.38	EGR1	9.85
ITGA3	2.75	BMP7	2.46	FGF23	2.30	IL18	3.73	FOS	59.89
ITGA8	-2.06	CSF1	-4.44	FGF6	4.76	IL1A	-8.00	MAP2K6	8.28
MMP10	-6.68	CSF2	-22.63	FGF7	-14.93	IL1B	-42.22	MAP4K1	4.92
MMP12	6.32	CSF3	-17.75	FGF9	3.25	IL2	6.06	MAPK6	5.86
MMP13	2.51	CXCL1	-115.36	GDF10	2.30	SPP1	3.36	MAPK8IP2	10.58
		ECGF1	-12.13			TGFB1	-3.86		
		EREG	-26.91			VEGFA	-5.86		
						VEGFC	-2.38		

Values of relative increase or decrease in gene expression represent the mean of two independent RT-Profiler experiments and were normalised using an experimental internal index of housekeeping genes. All expression values above 2 and below -2 were considered *per *definition as significantly modulated compared with unstrained basal control expression of the respective gene on each RT-Profiler.

Briefly, genes displaying up-regulated transcription levels on the MAP-kinase signalling and cell cycle profiler, allocate cell cycle promotion and inhibition as well (Figure 5, green columns). While cell cycle promoting genes included MAPK6, a member of the Ser/Thr protein kinase family, MAP4K1 which plays a role in the response to environmental stress, MAP2K6 which phosphorylates p38 stress kinase, and cyclins A1 and B1 (CCNA1 and CCNB1), genes associated with cell cycle inhibition enclosed CDKN2A, an inhibitor of CDK4-kinase, and CDKN2C, a member of the INK4A family of cyclin-dependent kinase inhibitors. A further gene, displaying up-regulation on this array was FOS which dimerises with JUN to form the AP-1 transcription factor complex, involved in proliferation, differentiation and transformation.

Concerning the ECM and integrin profiler (Figure 5, blue columns), elevated transcription was observed for integrin α3, facilitating focal contacts, and the matrix metalloproteinases MMP-12, and MMP-13. While MMP-12 degrades elastin and osteopontin (bone sialoprotein/SPP1), a matrix-associated molecule first identified in osteoblasts, substrates of MMP-13 include collagen.

Genes up-regulated on the growth factor array (Figure 5, orange columns) enclosed members of the mitogenic and cell survival promoting FGF family, explicitly FGF-6, FGF-9, and FGF-14, and members of the BMP-/TGF-β-(super) family, particularly BMP-4 and BMP-7 which share bone inductive abilities, and GDF-8, regulating cell growth and differentiation. In addition, amplified transcription was also noted for IL-2 the master molecule for B and T-lymphocyte proliferation.

An unexpected finding was that none of the down-regulated genes assigned to the MAP-kinase signalling profiler, suggesting that genes associated with cell cycle and its control are mechano-sensitive in a non-negative transcriptional fashion. The ECM and integrin array (Figure 5, blue columns) revealed reduced transcription for MMP-10 which cleaves proteoglycanes and fibronectin, and for the collagens (COL) COL-1 and 14, representing important constituents of the periodontal ECM. Genes down-regulated on the growth factor array (Figure 5, orange columns) belong to the group of colony stimulating factors and neutrophile activating proteins, controlling the production, differentiation, and function of granulocytes (CSF2, CSF3, and CXCL1), but also to the angiogenic factors, represented by VEGF and ECGF1 (endothelial cell growth factor). Further growth factors whose transcription was negatively affected by 30 minutes of strain were EREG (epiregulin), a member of the EGF family, IL-1β, an important cytokine in inflammatory response as well as FGF members FGF-7 and FGF-2, and BMP members BMP-1 and BMP-2, respectively.

## Discussion

In cells of solid tissues, focal contact integrins render key molecules in mechano-perception and are initial elements in mechano-transduction. Our analysis reveals that they appear also mechano-responsive, since mechanical strain reinforced expression of focal contact integrin subunits β1 and β3. Although not investigated on the mRNA level, PDL cells have been shown to up-regulate β1 integrin upon strain [25], thereby rendering a transcriptional equivalent to the protein situation described above. Moreover, in a previous study we have detected a strain-dependent topographical re-distribution of the β1 protein in PDLF [9], hence supporting the eventual integrin mechano-sensitivity. At intracellular sites, the non-coherent fashion by which the protein amount and the activation of the mechano-signalling kinase FAK/p125^FAK ^are modulated in PDL cells point to a temporal independent regulation, indicating that induction of mechano-transduction occurs as an early strain response. In parallel, early activation of FAK/p125^FAK ^upon strain application has been described for osteoblast-like cells [26], which, similar to PDL cells, are capable to form mineralised tissue. In the context of interrelationships between the molecular constituents of the focal adhesion complex and the actin cytoskeletal component, as evidenced by Geiger and co-workers [20], neither the applied strain intensity nor the duration displayed obvious impact on the protein abundance in PDLF. This observation suggests strain-independence concerning the cellular actin amount, though our protein detection by immunoblot can not exclude strain-associated spatial actin reallocations.

Similar to FAK/p125^FAK^, also the MAP-kinases p42/44^ERK ^and p38 stress-kinase revealed disconnection concerning modulation of protein quantity and phosphorylation/activation at the given strain periods. This finding shows on the one hand that modulation of intracellular signalling occurs uncoupled from the protein amount and on the other hand that both protein features of the kinases under study are addressed by mechanical forces. In addition, the strain-derived activation of all three kinases strongly suggests that they are also key players in mechano-transduction in PDL cells, and in this context may be down-stream located or even down-stream targets of FAK/p125^FAK^. With respect to p42/44^ERK ^this assumption is backed up by findings elaborated in osteoblastic cells which demonstrated that cell transfection with mutated FAK completely blocked ERK^2^-phosphorylation [26]. Further evidence for ERKs as mechano-responding signalling molecules is provided by other studies on mesangial cells and myocytes which demonstrated ERK-activation preceding strain application [22,23]. In addition, the latter study also affirmed a putative cross-talk between p42/44^ERK ^and p38 stress-kinase by demonstrating that in myocytes blockade of p38 activation completely inhibits ERK-phosphorylation [23]. Concerning the phosphorylation of p38 with matched p42/44^ERK ^in our PDL cells, the evidence of stronger p38 activation at 15 minutes upon strain appears to be a sign of higher sensitivity and therefore, higher mechano-responsiveness of stress-kinase at early stages of mechano-transduction.

By employing inhibitors specifically directed against p42/44^ERK ^and p38 stress-kinase phosphorylation, we demonstrated severe decline in the activation of these kinases, thereby truly proving their involvement in PDL-innate mechano-transduction. Concomitant with the up-regulation of p42/44^ERK ^and p38 stress-kinase in particular, we have detected increased protein levels for activated MMP-13 already at 15 minutes of strain, a time period allocating this protease also as an early mechano-responsive molecule. As a consequence arising from this prompt up-regulation of activated MMP-13, an early strain-dependent perturbation of ECM homeostasis through matrix degradation appears possible. Against this background it should be noted that we could not detect higher protein levels for one of the most striking MMP-inhibitors, TIMP-1 at any strain period tested. Additional support for the above-mentioned hypothesis comes from a study in osteoblasts which revealed elevated levels of ECM-degrading MMP gene expression with emphasis on MMP-1 and MMP-3, while TIMP-1 and TIMP-2 remained fairly unchanged [27]. Under the action of the MAP-kinase-specific inhibitors, expression of activated MMP-13 displayed significant cessation, a finding which demonstrates a mechanistic causality between p42/44^ERK ^and p38 stress-kinase activity as well, and the biological active form of the protease MMP-13 for PDL cells. With respect to the contrarious situation seen for the inhibitor-driven decrease of MAP-kinase activity and the reduction of MMP-13 expression, a superior priority of the p42/44^ERK ^mechano-transduction pathway for MMP-13 expression can be speculated. The pivotal role of p42/44^ERK ^for MMP expression can also be deduced from the previously cited study by Jansen and co-workers who found serious reduction in MMP-1 and MMP-3 gene expression upon usage of ERK-specific inhibitors [27].

Application of pathway-specific RT profiler arrays assigning to the biological functions MAP-kinase signalling and cell cycle, ECM and integrins, and growth factors revealed striking modulation of gene transcription on all arrays at 30 minutes. This modulation included up- and down-regulation of genes, thereby indicating that cellular gene transcription is affected earlier compared to most of the proteins investigated in this study. Concerning elevation of pathway-specific genes, the signalling molecule MAPK6 fits to the concept of being stress-associated, since from the cellular viewpoint application of mechanical forces can be considered as environmental stress. Further, up-regulation noted for MAP4K1 appears likely due to its function to phosphorylate p38 stress-kinase, a process which has been detected oscillating in the strained PDL cells. In case of transcription factors involved in signalling processes, the FOS gene as constituent of the AP-1 transcription complex was enhanced. This is an interesting finding, since the promoter of MMP-13 which has been part of this study contains an AP-1 binding element [28]. With respect to matrix and integrins, α3 displayed stronger transcription and represents an integrin subunit which in conjunction with β1 constitutes focal contacts [29]. On the part of MMPs, increase denoted for MMP-12 and MMP-13 also points to matrix and particularly elastin and collagen degradation as a response of PDLF to strain exposure. This process appears possible, since PDL cells synthesise tropoelastin [30], and *in vitro *studies on other periodontal cells have shown that gingival fibroblasts expressed higher levels of MMP-12 following bacterial challenge [31]. Matrix-associated molecules such as osteopontin which are immanent characteristics for PDL cells were also found to be enhanced upon strain, and may here contribute to emphasise features of mineralised tissues observed in these cells after mechanical loading [32]. The PDL cell's innate feature of expressing markers associated with mineralised tissues, e.g. bone is also reflected by the strain-induced higher levels for several fibroblast growth factor members, i.e. FGF-6, FGF-9 and FGF-14, and the bone inductive factors BMP-4, and BMP-7, which in sum belong to the BMP-/TGF-β superfamily [33]. Further, increase of IL-2, a cornerstone in inflammation and immunoresponse was noted which in the periodontium may indicate turn-over processes driven by mechanical forces, since it was detected at high levels in the crevicular fluid during orthodontic tooth movement [34]. In this context, the observed down-regulation of IL-1β another key molecule in inflammation may suggest a minor involvement of this cytokine in PDL cells subjected to mechanical strain.

With focus on the MAP-kinase signalling genes, none of them was involved in strain-dependent down-regulation, suggesting that transcription of mechano-sensitive cell cycle-associated genes is not negatively regulated. However, it has to remain open whether mechanical strain favours promotion or inhibition of the cell cycle in the PDL cells, since our transcription analysis revealed that genes of both functions displayed up-regulation. In addition, cell cycle arrest can not be excluded, since EREG a member of the proliferation-associated EGF family [35] was negatively affected. Down-regulated matrix molecules include collagens, COL-1 and COL-14, representing ECM substrates cleaved by MMP-13 [36]. Behind this transcriptional status for the collagens, MMP-13 showed elevation in the PDL cells on both the transcriptional and the protein level, and hereby emphasises the possibility for disruption of ECM homeostasis during application of mechanical forces.

## Conclusions

In the light of the findings elaborated from the protein and gene transcription analyses performed in this study on periodontal ligament fibroblasts subjected to mechanical strain, FAK/p125^FAK ^-mediated mechano-transduction identifies activated states of MAP-kinases p42/44^ERK ^and p38-stress kinase as a mechanism for MMP-13 expression, thereby indicating the mechanistic contribution of mechano-transducing molecules on executioners of ECM homeostasis.

## Methods

### Cell culture and strain application

Primary PDL fibroblasts (PDLF) were derived from the ligament tissues of periodontally healthy, non-carious human premolar teeth, extracted from juvenile donors (12-14 years) for orthodontic reasons with informed consent, and this study was approved by the institutional ethic committee of the Medical Faculty, University of Heidelberg (Vote number 148/2003; renewal 30.09.2005). Small tissue fragments were established as explant cultures by means of DME medium (PAA, Cölbe, Germany) supplemented with 10% foetal calf serum (FCS; Biochrom, Berlin, Germany), 2 mM L-glutamine (Invitrogen, Karlsruhe, Germany), and antibiotics (kanamycin, 50 mg/ml; Roche, Mannheim, Germany). After nearly reaching confluence, cells were used for strain experiments between passages 8 and 12. Strain application was carried out according to the method described by Hasagawa [37]. Briefly, following trypsination, 3.5 × 10^3^/cm^2 ^PDL cells were seeded on flexible-bottomed dishes (Greiner Bio-One, Frickenhausen, Germany), coated with coating medium, and grown until near-confluence. As an approach to the composition of the extracellular matrix (ECM) environment of PDLF *in vivo*, the coating medium in addition to 1% BSA (Sigma, Munich, Germany) comprises 20 μg/ml native collagen type-I (IBM, Leipzig, Germany) and 10 μg/ml fibronectin (Biomol, Hamburg, Germany). Both molecules are essentially found in the PDL's ECM [38]. The bottom of each dish was strained by induction of a continuous average strain of 2.5% [37] for periods of 0.25, 0.5, 1, 3, and 6 hours concerning the western blot experiments, and 0.5, 3 and 6 hours in case of RT-Profiler qPCR experiments, respectively. Irrespective from the *modus operandi*, unstrained cells served as controls. Concerning the forces acting on the PDLF, the continuous stretch mimics forces which are applied during orthodontic tooth movement using fixed appliances. The chosen time periods, mentioned above, reflect initial stages of therapeutically applied mechanical forces. Depending on the type of tooth and the type of tooth movement, orthodontic forces in a range of 0.15 - 2.5 Newton (N) are usual. Although this force range is of therapeutic significance, it is noteworthy to mention that it is not possible up till now to predict neither the exact force which acts on the periodontium on the single cell level nor the optimal force for an individual tooth [39]. Concerning the forces used in our study, in a first approach, translation of the average strain of 2.5% onto the single cell level reveals a force amount of approximately 30 nN.

For MAP-kinases inhibition experiments, PDL cells were additionally treated with protein kinase-specific inhibitors, SB202190 for phosphorylation inhibition of phosphor-p38 and UO-126 for specific inhibition of phospho-p42/44. Both inhibitors were purchased from Calbiochem (Merck, Darmstadt, Germany). PDL cells were incubated with U0126 (10 μM) or SB202190 (10 μM) for 1 h prior to strain application. After the incubation period, fresh DMEM medium was added to the cell cultures followed by strain application for the above mentioned time periods.

### RNA-isolation and quantitative RT-PCR-Profiler

Total *RNA *was isolated from nearly confluent human PDL cells from lumox dishes (Greiner bio-one, Frickenhausen, Germany) after periods of 0.5, 3, and 6 hours strain, by using the RNasy system (Qiagen, Hilden, Germany), and treated with RNase-free DNase (Qiagen, Hilden, Germany) for 15 min at room temperature (24°C). RNA concentration was measured with Bio-Rad electrophoresis system (Experion™ System, Bio-Rad, Munich, Germany) according to the manufacturer's instructions. Total RNA (1 μg) was reverse-transcribed into first strand cDNA using RT^2 ^First Strand Synthesis Kit (C-03) (SA Bioscience Corporation, Frederick, MD, USA) according to the manufacturer's instructions. cDNA was mixed with instrument-specific and ready-to-use RT^2^-qPCR master mix (RT^2^-SYBR^® ^Green/Fluorescein qPCR master mix, SA Bioscience Corporation, Frederick, MD, USA), and 25 μl of the master mix were pipetted into each well containing pre-dispensed gene-specific primer pairs. The pathway-specific qPCR experiments were performed for human extracellular matrix and integrins, human growth factors, and MAP-kinase signalling and cell cycle pathways (SA Biosciences Corporation, Frederick, MD, USA) and amplification was done after an initial cycle of 10 min at 95°C to activate the HotStart DNA polymerase, followed by the manufacture's specific PCR protocol. The annealing conditions for the pre-validated and pre-dispersed primer pairs were set to 55°C according to the manufacture's instructions. Relative gene expression levels were analysed using a modification of the ΔΔ*C*_T _equation, which allows counting for differences in efficiencies (*E *= 10^1/slope^) between the PCR reactions [40]. The Δ*C*_T _values were calculated using excel plug-in provided with the pathway-specific profilers (SA Biosciences Corporation, Frederick, MD, USA). The data were obtained from two individual experiments and normalised to the *C*_T _of the experimental internal set of 4 different housekeeping genes (B2M, HPRT1, RPL13A, GAPDH, ACTB). The relative expression levels were subjected to means *t*-test and only *p*-values less than 0.01 were considered statistically significant and plotted in Figure 5.

### Immunofluorescence (IIF)

After strain application, PDL cells were washed with ice cold PBS, fixed for 5 min with ethanol (96%, -20°C), and subsequently air-dried. For antibody treatment flexible membranes were cut in pieces and separated into inner and outer membrane parts. Thereafter, alcohol-fixed membrane pieces were incubated overnight with the primary antibodies directed against integrin subunits β1 and β3 (both mouse monoclonal, (wd) 1:50 in PBT (PBS containing 0.5% BSA, 0.5 Tween-20, and 0.02% NaN3), Santa Cruz Biotechnology, Heidelberg, Germany). Then, membrane pieces were washed in PBS for three times (5 min each), followed by incubation with the secondary fluorochrome-conjugated antibody (Alexa FluorTM 488, Mo Bi Tec Göttingen, Germany, IgG (H+L) goat anti-mouse, wd 1:100) for 1 hour at room temperature. To allow for nucleus counterstain, DAPI-staining (Sigma, Deisenhofen, Germany; wd 1:1000) was performed for 10 min at RT. After air drying, membrane pieces were embedded in mounting medium ProlongGold antifade reagent (Invitrogen, Karlsruhe, Germany), and documented by using a Leica digital camera (DFC300 FX), and the fluorescence microscope with Leica Application Suit 2.4.0 software (DMRE, Leica TCS/NT, Leica, Bensheim, Germany). All images were taken with equal exposure times within the experimental set up for each antibody.

### Western blot analysis

After strain periods of 0.25, 0.5, 1, 3, and 6 hours, cells were washed with PBS and lysed on ice with cold RIPA buffer (Sigma-Aldrich, Steinheim, Germany) containing protease inhibitors (Complete Mini, Roche Diagnostics, Mannheim, Germany). After centrifugation (8000 g, 10 min), protein amounts were measured with the experion system (Experion™ Pro260, Pro260 Chips, Bio-Rad Laboratories, Munich, Germany) according to manufacturer's instructions. In all western blot experiments equal protein aliquots of 25 μg of protein were diluted in NuPAGE LDS sample buffer and NuPAGE sample reducing agent (Invitrogen, Karlsruhe, Germany), heated at 70°C for 5 min and separated by NuPAGE 4-12% Bis-Tris gradient gels (Invitrogen, Karlsruhe, Germany). For immunoblotting, the separated proteins were transferred onto a PVDF membrane (Invitrolon™ PVDF Filter Paper Sandwich, Invitrogen, Karlsruhe, Germany) in a Trans-Blot electrophoretic transfer cell (BioRad, Munich, Germany). Subsequently, immunodetection was performed by incubation the PDVF membranes with primary antibodies directed against focal adhesion kinase (FAK), phospho-specific FAK^Tyr576 ^(rabbit monoclonal, (wd) 1:1000 and 1:500 Santa Cruz Biotechnology, Heidelberg, Germany), MMP-13 (mouse monoclonal, (wd) 1:1000, RD Systems, Wiesbaden-Nordenstadt, Germany), p44/42 MAP-kinase, phospho-specific p44/42 MAP-kinase ^(Thr202/Tyr204) ^(rabbit polyclonal, (wd) 1:1000 and 1:500, Cell Signalling Technology, Danvers, MA, USA), p38 MAP-kinase, phospho-specific p38 MAP-kinase^(Thr180/Tyr182) ^(rabbit monoclonal, (wd) 1:1000 and 1:500, Epitomics, Burlingame, CA, USA), and integrin β3 (mouse monoclonal, (wd) 1:1000, Santa Cruz Biotechnology, Heidelberg, Germany) in PBT for one hour at room temperature. Specific proteins were revealed by the WesternBreeze Chromogenic Immunodetection System (Invitrogen, Karlsruhe, Germany). The developed blots were documented by a digital camera (Casio Digital Camera EX-P700, Casio Europe GmbH, Norderstedt, Germany) and images were analysed using the image analysis software (BioDocAnalyzer 2.1, Biometra, Goettingen, Germany) for quantification of different band gray pixel values. To exclude that the observed changes in band intensities might be within the experimental noise range, analysis was performed by direct import of the digital image of each blot into the software, thereby subtracting the background with a standard algorithm to minimise experimental noise ratio. The gray pixel values of modulated protein expression following strain application were always normalised with the expression of unstrained control PDL cells, and plotted into graphs. To get a maximum of reproducibility, the experimental western blot setup was standardised concerning the protein amount loaded on the gel, equal time period for development of the western blot membranes with substrate and the same camera documentation setup, which includes the camera-membrane distance and the exposure time. This standardised western blot protocol allows detection of band intensity changes in independent experiments below 10%. Data of the western blot experiments represent the mean of three independent experiments (n = 3, mean ± SD), and for proper statistical analysis the means were subjected to the Students T-test (MedCalc^® ^9.0.1.1, Mariakerke, Belgium). Hereby, all compared mean values with p < 0.01 were denoted as statistical significant (Table 1) and were marked with an asterisk in the plotted graphs.

## Authors' contributions

NZ performed the main experimental part of the study. AA helped to design the study and contributed with his expertise to the discussion of cell signalling pathways. TS participated in the design of the study, supervised the experimental part of the investigations and performed the data evaluation and graphical outline of the manuscript. DW contributed to the experimental part of the manuscripts with westernblot analyses. AK performed the cell culture experiments. EM and SS performed the densitometric evaluation of the western blots. PT participated in the design of the study, wrote the manuscript and contributed with his expertise in discussions concerning the successful finalization of the investigations. All authors read and approved the final version of the manuscript

## References

[B1] von Bohl M Kuijpers-Jagtman AM Hyalinization during orthodontic tooth movement: a systematic review on tissue reactions Eur J Orthod 2009 31 30 36 10.1093/ejo/cjn080 19073957

[B2] Bartold PM McCulloch CA Narayanan AS Pitaru S Tissue engineering: a new paradigm for periodontal regeneration based on molecular and cell biology Periodontol 2000 24 253 269 10.1034/j.1600-0757.2000.2240113.x 11276871

[B3] Cho MI Garant PR Development and general structure of the periodontium Periodontol 2000 24 9 27 10.1034/j.1600-0757.2000.2240102.x 11276876

[B4] Tsuruga E Sato A Ueki T Nakashima K Nakatomi Y Ishikawa H Yajima T Sawa Y Integrin alphavbeta3 regulates microfibril assembly in human periodontal ligament cells Tissue Cell 2009 41 85 89 10.1016/j.tice.2008.07.005 18789468

[B5] Himmel M Ritter A Rothemund S Pauling BV Rottner K Gingras AR Ziegler WH Control of high affinity interactions in the talin C-terminus - how talin domains coordinate protein dynamics in cell adhesions J Biol Chem 2009 284 13832 13842 10.1074/jbc.M900266200 19278997 PMC2679484

[B6] Muller EJ Williamson L Kolly C Suter MM Outside-in signaling through integrins and cadherins: a central mechanism to control epidermal growth and differentiation? J Invest Dermatol 2008 128 501 516 10.1038/sj.jid.5701248 18268536

[B7] Papusheva E de Queiroz FM Dalous J Han Y Esposito A Jares-Erijmanxa EA Jovin TM Bunt G Dynamic conformational changes in the FERM domain of FAK are involved in focal-adhesion behavior during cell spreading and motility J Cell Sci 2009 122 656 666 10.1242/jcs.028738 19208768

[B8] Roy S Ruest PJ Hanks SK FAK regulates tyrosine phosphorylation of CAS, paxillin, and PYK2 in cells expressing v-Src, but is not a critical determinant of v-Src transformation J Cell Biochem 2002 84 377 388 10.1002/jcb.10025 11787067

[B9] Molina T Kabsch K Alonso A Kohl A Komposch G Tomakidi P Topographic changes of focal adhesion components and modulation of p125FAK activation in stretched human periodontal ligament fibroblasts J Dent Res 2001 80 1984 1989 10.1177/00220345010800110701 11759007

[B10] Sturgill TW MAP kinase: it's been longer than fifteen minutes Biochem Biophys Res Commun 2008 371 1 4 10.1016/j.bbrc.2008.04.002 18406346

[B11] Gaestel M Specificity of signaling from MAPKs to MAPKAPKs: kinases' tango nuevo Front Biosci 2008 13 6050 6059 10.2741/3136 18508642

[B12] Rauch C Loughna PT Stretch-induced activation of ERK in myocytes is p38 and calcineurin-dependent Cell Biochem Funct 2008 26 866 869 10.1002/cbf.1518 18956431

[B13] Fulton DL Sundararajan S Badis G Hughes TR Wasserman WW Roach JC Sladek R TFCat: the curated catalog of mouse and human transcription factors Genome Biol 2009 10 R29 10.1186/gb-2009-10-3-r29 19284633 PMC2691000

[B14] Haasper C Jagodzinski M Drescher M Meller R Wehmeier M Krettek C Hesse E Cyclic strain induces FosB and initiates osteogenic differentiation of mesenchymal cells Exp Toxicol Pathol 2008 59 355 363 18222075 10.1016/j.etp.2007.11.008

[B15] Sakai H Urasawa K Oyama N Kaneta S Saito T Kitabatake A Tsutsui H Induction of c-fos mRNA expression by pure pressure overload in cultured cardiac myocytes Int Heart J 2007 48 359 367 10.1536/ihj.48.359 17592200

[B16] Song G Ju Y Shen X Luo Q Shi Y Qin J Mechanical stretch promotes proliferation of rat bone marrow mesenchymal stem cells Colloids Surf B Biointerfaces 2007 58 271 277 10.1016/j.colsurfb.2007.04.001 17499488

[B17] Tan TW Yang WH Lin YT Hsu SF Li TM Kao ST Chen WC Fong YC Tang CH Cyr61 increases migration and MMP-13 expression via alphavbeta3 integrin, FAK, ERK and AP-1-dependent pathway in human chondrosarcoma cells Carcinogenesis 2009 30 258 268 10.1093/carcin/bgn284 19126648

[B18] Embery G Waddington RJ Hall RC Last KS Connective tissue elements as diagnostic aids in periodontology Periodontol 2000 24 193 214 10.1034/j.1600-0757.2000.2240109.x 11276867

[B19] Melendez-Zajgla J Del Pozo L Ceballos G Maldonado V Tissue inhibitor of metalloproteinases-4. The road less traveled Mol Cancer 2008 7 85 10.1186/1476-4598-7-85 19025595 PMC2599898

[B20] Geiger B Spatz JP Bershadsky AD Environmental sensing through focal adhesions Nat Rev Mol Cell Biol 2009 10 21 33 10.1038/nrm2593 19197329

[B21] Flier A van der Sonnenberg A Function and interactions of integrins Cell Tissue Res 2001 305 285 298 10.1007/s004410100417 11572082

[B22] Krepinsky J Mechanical stretch-induced signal transduction in cultured mesangial cells Methods Mol Biol 2009 466 205 221 19148608 10.1007/978-1-59745-352-3_15

[B23] Rauch C Loughna PT Stretch-induced activation of ERK in myocytes is p38 and calcineurin-dependent Cell Biochem Funct 2008 26 866 869 10.1002/cbf.1518 18956431

[B24] Leonardi R Talic NF Loreto C MMP-13 (collagenase 3) immunolocalisation during initial orthodontic tooth movement in rats Acta Histochem 2007 109 215 220 10.1016/j.acthis.2007.01.002 17350083

[B25] Bolcato-Bellemin AL Elkaim R Abehsera A Fausser JL Haikel Y Tenenbaum H Expression of mRNAs encoding for alpha and beta integrin subunits, MMPs, and TIMPs in stretched human periodontal ligament and gingival fibroblasts J Dent Res 2000 79 1712 1716 10.1177/00220345000790091201 11023268

[B26] Boutahar N Guignandon A Vico L Lafage-Proust MH Mechanical strain on osteoblasts activates autophosphorylation of focal adhesion kinase and proline-rich tyrosine kinase 2 tyrosine sites involved in ERK activation J Biol Chem 2004 279 30588 30599 10.1074/jbc.M313244200 15096502

[B27] Jansen JH Jahr H Verhaar JA Pols HA Chiba H Weinans H van Leeuwen JP Stretch-induced modulation of matrix metalloproteinases in mineralizing osteoblasts via extracellular signal-regulated kinase-1/2 J Orthop Res 2006 24 1480 1488 10.1002/jor.20186 16705736

[B28] Tan TW Yang WH Lin YT Hsu SF Li TM Kao ST Chen WC Fong YC Tang CH Cyr61 increases migration and MMP-13 expression via alphavbeta3 integrin, FAK, ERK and AP-1-dependent pathway in human chondrosarcoma cells Carcinogenesis 2009 30 258 268 10.1093/carcin/bgn284 19126648

[B29] Carter WG Kaur P Gil SG Gahr PJ Wayner EA Distinct functions for integrins alpha 3 beta 1 in focal adhesions and alpha 6 beta 4/bullous pemphigoid antigen in a new stable anchoring contact (SAC) of keratinocytes: relation to hemidesmosomes J Cell Biol 1990 111 3141 3154 10.1083/jcb.111.6.3141 2269668 PMC2116384

[B30] Redlich M Roos H Reichenberg E Zaks B Grosskop A Bar Kana I Pitaru S Palmon A The effect of centrifugal force on mRNA levels of collagenase, collagen type-I, tissue inhibitors of metalloproteinases and beta-actin in cultured human periodontal ligament fibroblasts J Periodontal Res 2004 39 27 32 10.1111/j.1600-0765.2004.00700.x 14687224

[B31] Zhou J Windsor LJ Porphyromonas gingivalis affects host collagen degradation by affecting expression, activation, and inhibition of matrix metalloproteinases J Periodontal Res 2006 41 47 54 10.1111/j.1600-0765.2005.00835.x 16409255

[B32] Wongkhantee S Yongchaitrakul T Pavasant P Mechanical stress induces osteopontin expression in human periodontal ligament cells through rho kinase J Periodontol 2007 78 1113 1119 10.1902/jop.2007.060433 17539726

[B33] Issa MJPTR Pitol DL Mello SAS TGF-[beta] and new bone formation Int J Morphol 2006 24 399 405

[B34] Basaran G Ozer T Kaya FA Hamamci O Interleukins 2, 6, and 8 levels in human gingival sulcus during orthodontic treatment Am J Orthod Dentofacial Orthop 2006 130 7 e1 6 10.1016/j.ajodo.2005.12.027 16849065

[B35] Zeng F Singh AB Harris RC The role of the EGF family of ligands and receptors in renal development, physiology and pathophysiology Exp Cell Res 2009 315 602 610 10.1016/j.yexcr.2008.08.005 18761338 PMC2654782

[B36] Hernandez M Martinez B Tejerina JM Valenzuela MA Gamonal J MMP-13 and TIMP-1 determinations in progressive chronic periodontitis J Clin Periodontol 2007 34 729 735 10.1111/j.1600-051X.2007.01107.x 17716308

[B37] Hasegawa S Sato S Saito S Suzuki Y Brunette DM Mechanical stretching increases the number of cultured bone cells synthesizing DNA and alters their pattern of protein synthesis Calcif Tissue int 1985 37 431 436 10.1007/BF02553714 3930042

[B38] Waddington RJ Embery G Proteoglycans and orthodontic tooth movement J Orthod 2001 28 281 290 10.1093/ortho/28.4.281 11709593

[B39] Diedrich P Praxis der Zanhheilkunde: Kieferorthopädie II 2000 Urban & Fischer, München, Jena

[B40] Livak KJ Schmittgen TD Analysis of relative gene expression data using real-time quantitative PCR and the 2(-Delta Delta C(T)) Method Methods 2001 25 402 408 10.1006/meth.2001.1262 11846609

